# *Chlamydia trachomatis* in Cervical Lymph Node of Man with Lymphogranuloma Venereum, Croatia, 2014^1^

**DOI:** 10.3201/eid2404.171872

**Published:** 2018-04

**Authors:** Branimir Gjurašin, Snježana Židovec Lepej, Michelle J. Cole, Rachel Pitt, Josip Begovac

**Affiliations:** University Hospital for Infectious Diseases Dr. Fran Mihaljević, Zagreb, Croatia (B. Gjurašin, S.Ž. Lepej, J. Begovac);; Public Health England, London, UK (M.J. Cole, R. Pitt);; University of Zagreb School of Medicine, Zagreb (J. Begovac)

**Keywords:** Chlamydia trachomatis, bacteria, genovar, variant L2b, lymphogranuloma venereum, LGV, buboes, cervical lymph node, proctitis, HIV infection, men who have sex with men, MSM, Croatia

## Abstract

We report an HIV-infected person who was treated for lymphogranuloma venereum cervical lymphadenopathy and proctitis in Croatia in 2014. Infection with a variant L2b genovar of *Chlamydia trachomatis* was detected in a cervical lymph node aspirate. A prolonged course of doxycycline was required to cure the infection.

*Lymphogranuloma venereum* (LGV) is a sexually transmitted infection caused by serovars L1, L2, and L3 of the bacterium *Chlamydia trachomatis*. The infection typically causes genital ulcers, proctitis, or femoral/inguinal lymphadenopathy with or without constitutional symptoms. In the past decade, outbreaks of LGV have been reported in North America, Australia, and Europe, mainly as proctitis among HIV-infected men who have sex with men (MSM) (*1*). We report a patient with pharyngitis, proctitis, and cervical lymphadenitis in whom LGV-specific DNA was detected by real-time reverse transcription PCR (RT-PCR) in a cervical lymph node fine-needle aspirate.

The patient was a 48-year-old, HIV-positive man in Croatia who came to an outpatient HIV clinic in August 2014 with perianal pain for 10 days and bloody rectal discharge with normal stool consistency. He also reported a painful, enlarged cervical lymph node but did not have a sore throat. On the first day of the illness, he had fever, which subsided the next day. He reported having unprotected receptive anal and oral sex with other men while visiting Berlin, Germany, 2 weeks earlier. Clinical examination demonstrated exudate on the right tonsil, a painful and enlarged right cervical lymph node (5 × 2 cm) (Technical Appendix Figure), perianal pain on palpation, and a purulent rectal discharge.

The patient was given a diagnosis of HIV infection in 2002 and had been receiving antiretroviral therapy since July 2002. Plasma viremia had been undetectable since October 2002, and his CD4+ T-cell count before this illness was 2,082 cells/mm^3^. His clinical history included treatment for neurosyphilis, epilepsy, and diarrhea caused by *Microsporidiae* spp., *Blastocystis hominis*, and *Entamoeba histolytica*.

During examination at the HIV clinic, specimens were obtained from the pharynx, rectum, and urine for culture and a nucleic acid amplification test (NAAT). During fine-needle aspiration of a cervical lymph node, ≈1 mL of pus was removed and analyzed. The lymph node aspirate and a rectal swab specimen were positive for *C. trachomatis* DNA by the *C. trachomatis*/*Neisseria gonorrhoeae* RT-PCR (Abbott Laboratories, Abbott Park, IL, USA).

Cytologic examination of the fine-needle aspirate of the affected lymph node predominantly showed elements of granulomatous inflammation. An indirect immunofluorescence assay serum test result for *C. trachomatis* antibodies was positive (IgG titer >1:512, IgA titer 1:256). Test results for *N. gonorrhoeae* were negative (culture of the rectal swab and NAAT for urine and rectum). Results of a throat culture for *Streptococcus pyogenes* and routine lymph node aspirate culture for bacteria were also negative. Serum serologic test results were negative for acute infection with *Treponema pallidum, Bartonella* spp., and *Toxoplasma gondii*.

DNA from the lymph node specimen was frozen and sent to Public Health England (London, UK) in February 2017. LGV-specific DNA was detected by using an in-house TaqMan RT-PCR. The sequence of the outer membrane protein gene from lymph node punctate was identical to that of the *C. trachomatis* L2 reference strain L2/434/Bu.

At the initial visit, the patient was treated with intravenous ceftriaxone (2 g) and oral doxycycline (2 × 100 mg). After NAATs showed *C. trachomatis* infection, only doxycycline therapy was continued. Symptoms of proctitis subsided in 2 days. However, because cervical lymphadenitis persisted, we treated the patient with a prolonged course (6 weeks) of doxycycline. Eventually, the patient showed a full recovery.

Our report indicates that LGV might be present in MSM in Croatia. The first NAAT-confirmed case of LGV in southeastern Europe was reported in Slovenia and described an HIV-negative MSM who was ill in 2015 (*2*). LGV is probably underdiagnosed in southeastern Europe because of lack of diagnostics and awareness of the infection.

There have been only a few case reports of LGV with associated cervical lymphadenopathy (*3**–**8*) (Table). Some cases had generalized lymphadenopathy (axillar, supraclavicular, and retroperitoneal) with constitutional symptoms (*3*); pharyngitis/odynophagia/proctitis/tongue soreness (*4*,^7^); constitutional symptoms (*5**,**7*); tonsillitis (*6*); or skin lesions (*8*). Case reports have also been described of LGV with supraclavicular and mediastinal lymphadenopathy without cervical involvement (*9*). In all of these cases, infection with LGV caused by *C. trachomatis* was established by serologic testing or an NAAT for a pharyngeal specimen. It is essential to maintain a high level of clinical suspicion for LGV in MSM even if noninguinal/femoral lymph nodes are affected.

**Table:** Characteristics of 8 patients with lymphogranuloma venereum and cervical lymphadenopathy*

Reference	Patient age, y/sex	Clinical presentation	Method of laboratory conformation	Therapy/duration
Andrada et al. (*4*)	30/M	Mouth ulcer, weight loss, cervical lymphadenopathy	Serologic analysis	Tetracycline/5 wk
Thorsteinsson et al. (*3*)	31/M	Fever, supraclavicular, axillar, retroperitoneal, and cervical lymphadenopathy	Serologic analysis	Tetracycline/4 wk
Watson et al. (*6*)	19/F	Sore throat, tonsillitis, arthritis, erythema nodosum, cervical lymphadenopathy	Serologic analysis	Phenoxymethylpenicillin, indomethacin, erythromycin†
Albay and Mathison (*5*)	18/F	Fever, cervical lymphadenopathy	Serologic analysis	Ampicillin/sulbactam, doxycycline†
Tchernev et al. (*8*)	36/M	Facial skin lesions, cervical and axillary lymphadenopathy	NAAT: *Chlamydia trachomatis* DNA in skin lesions and serologic analysis	Surgical excision of cervical lymph nodes; pentamidine and doxycycline/3 wk
Dosekun et al. (*4*)	32/M	Sore throat, cervical lymphadenopathy, odynophagia, mouth ulcer, proctitis, cervical lymphadenopathy	NAAT: LGV-specific DNA in pharyngeal swab specimen	Amoxicillin/1 wk, doxycycline/1 wk
Dosekun et al. (*4*)	27/M	Sore throat, cervical lymphadenopathy, odynophagia, mouth ulcer, proctitis, cervical lymphadenopathy	NAAT: LGV-specific DNA in pharyngeal and rectal swab specimens	Azithromycin/1 g, doxycycline/2 wk
This study	48/M	Fever, cervical lymphadenopathy, proctitis	NAAT: LGV-specific DNA in cervical lymph node sample obtained by fine-needle aspirate; serologic analysis	Ceftriaxone/5 d, doxycycline/6 wk

*LGV, lymphogranuloma venereum; NAAT, nucleic acid amplification test. †Duration of therapy not reported.

The recommended treatment for LGV is doxycycline for 21 days. However, several clinical observations have suggested that a 21-day course of doxycycline therapy is insufficient for treating inguinal bubonic LGV (*2**,**10*), Recommendations have been given to carefully follow up with patients and continue doxycycline treatment until symptoms resolve (*10*). We followed these recommendations for our patient who had bubonic cervical lymph node LGV.

Technical Appendix Additional information on *Chlamydia trachomatis* in cervical lymph node of man with lymphogranuloma venereum, Croatia 2014.
