# Supplementary material for: Chlamydia trachomatis in Cervical Lymph Node of Man with Lymphogranuloma Venereum, Croatia, 2014
Source: Emerg Infect Dis. 2018 Apr;24(4):806–8. doi: 10.3201/eid2404.171872 (PMC5875274; doi:10.3201/eid2404.171872)
Supplement: Technical Appendix — Additional information on Chlamydia trachomatis in cervical lymph node of man with lymphogranuloma venereum, Croatia 2014. [file 17-1872-Techapp-s1.pdf]

Article DOI: https://doi.org/10.3201/eid2404.171872

Chlamydia trachomatis in Cervical Lymph Node of Man with Lymphogranuloma Venereum, Croatia 2014

Technical Appendix.

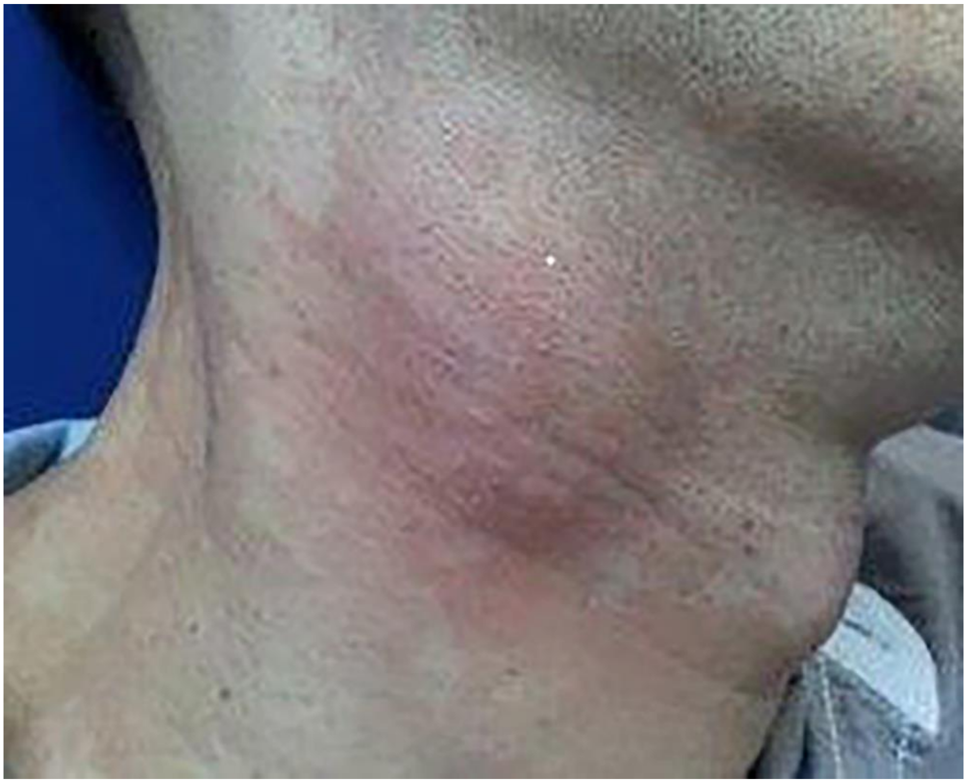

Technical Appendix Figure. Right cervical lymphadenopathy in 48-year-old man with lymphogranuloma venereum, Croatia 2014.
